# Allergic Rhinitis: From Pathology to Novel Therapeutic Approaches

**DOI:** 10.3390/biomedicines14092029

**Published:** 2026-09-09

**Authors:** Shiwang Tan, Shaoqing Yu

**Affiliations:** 1 Department of Otolaryngology, Head and Neck Surgery, Tongji Hospital, School of Medicine, Tongji University, Shanghai 200065, China; 2 Department of Allergy, Tongji Hospital, School of Medicine, Tongji University, Shanghai 200065, China

Allergic rhinitis (AR) is a common chronic inflammatory disorder of the upper airway in which allergen sensitization interacts with epithelial, neural, and immune responses, as well as comorbidity and treatment context, to shape clinical expression. Current practice parameters place intranasal pharmacotherapy and allergen immunotherapy (AIT) within individualized management strategies [1], while emerging evidence distinguishes rhinitis alone from rhinitis–asthma multimorbidity on the basis of clinical, sensitization, molecular, and treatment-response characteristics [2]. Against this broader clinical and biological background, the five contributions to this Special Issue address neuroimmune signaling, adaptive immune regulation, transcriptomic heterogeneity, age-related variation in AIT, and real-world biologic treatment. The resulting scope extends from symptom generation and immune regulation to molecular characterization and therapeutic response, reflecting the closer integration of clinical phenotype and tissue biology in contemporary AR research.

The nasal epithelium forms the primary interface between inhaled exposures and the local airway environment. Its structural, chemical, immune, and microbiological components contribute to mucosal homeostasis, while disruption of the epithelial barrier can increase allergen access and modify inflammatory signaling and communication with sensory and immune cells [3]. These interactions place the epithelium at the intersection of environmental sensing, neural activation, and local immune responses in AR.

Nasal hyperreactivity represents an important link between environmental stimuli and symptom generation. Kang et al. reviewed the roles of the cold-sensitive transient receptor potential channels TRPA1 and TRPM8 in AR and chronic rhinosinusitis [4]. Cold exposure can activate sensory nerves through these channels, promoting neuropeptide release and local immune modulation and thereby linking sensory activation with immune responses in the nasal mucosa. TRPA1- and TRPM8-related signaling may contribute to persistent and stimulus-induced nasal symptoms, supporting further investigation of these pathways as potential therapeutic targets.

Adaptive immune regulation is another theme of the Special Issue. Luo et al. investigated the interleukin-37 (IL-37)–T follicular helper 2 (Tfh2) axis using blood samples from patients with AR, isolated-cell experiments, Tfh2/B-cell coculture, and an ovalbumin-induced mouse model [5]. IL-37 suppressed Tfh2-associated cytokine production, reduced IgE production by B cells, and ameliorated several features of experimental AR. Regulatory B cells contribute to a complementary arm of immune regulation in AR; Fan et al. reviewed their roles in allergic inflammation and the development of immune tolerance [6]. Tfh2 and regulatory B-cell responses thus highlight the balance between type 2 effector activity and regulatory mechanisms that shape allergen-specific immunity.

Disease characterization in AR increasingly extends from accessible airway biomarkers to transcriptomic and cell-resolved profiling. Yu et al. synthesized evidence on exhaled carbon monoxide (eCO) in asthma and AR, identifying distinct patterns across airway phenotypes, with higher eCO in asthma and values in AR closer to those observed in healthy controls [7]. At a different biological scale, Surita et al. integrated bulk and single-cell transcriptomic data to examine molecular and cellular heterogeneity in AR [8]. Their analyses identified immune-related transcriptional variation and differences in immune-cell composition and intercellular communication, while the single-cell data suggested reduced CD4+ central memory T-cell abundance and attenuated communication involving this population. An exploratory molecular classifier showed cohort-dependent performance, with strong discrimination in the training dataset and limited discrimination in external validation, while computational drug repositioning generated candidate compounds for experimental evaluation. Airway biomarkers and cell-resolved molecular data offer complementary levels of disease characterization, ranging from readily accessible phenotyping to the localization of immune alterations within specific cellular populations. Cross-cohort validation can further distinguish molecular features that reproduce across populations and analytical platforms.

AIT is an established disease-modifying treatment for allergen-driven AR. A randomized phase III trial demonstrated reductions in rhinitis symptoms and medication use with house dust mite sublingual immunotherapy in adults with moderate-to-severe disease [9]. Jin et al. examined age-related differences in clinical response to house dust mite subcutaneous immunotherapy (SCIT) in a retrospective cohort of 240 patients [10]. Nasal symptoms and quality-of-life scores improved across age groups, while response rates were highest in children and progressively lower in middle-aged and elderly patients. Peripheral regulatory T-cell and regulatory B-cell responses also differed by age. In a separate analysis of a public nasal single-cell dataset, age-associated T-cell senescence was observed. These parallel clinical and transcriptomic findings bring immune aging into the study of AIT response. Longitudinal studies that track T-cell and regulatory-cell states during SCIT alongside clinical outcomes could clarify how age-related immune trajectories relate to heterogeneity in treatment response across the life course.

Mechanistically, allergen-specific tolerance involves coordinated changes in regulatory T- and B-cell activity, suppressive cytokines such as IL-10 and TGF-β, antibody responses, and effector-cell activation [11]. Viewed in this context, the IL-37–Tfh2 findings of Luo et al., the regulatory B-cell studies summarized by Fan et al., and the age-stratified SCIT study of Jin et al. describe complementary components of immune regulation in AR. Longitudinal sampling during AIT could define how these cellular and humoral responses evolve with treatment and which immune trajectories accompany sustained clinical improvement. 

Increasingly, emerging theories have driven new therapeutic options for allergic rhinitis, such as stem cells [12], exosomes [13], and biologics. Biologic therapy has expanded the therapeutic landscape of type 2 airway inflammation. Omalizumab improved symptoms and quality of life in a randomized trial of seasonal AR [14], while stapokibart, an IL-4Rα-targeting monoclonal antibody, improved nasal and ocular symptoms and quality of life in a randomized phase III trial of adults with moderate-to-severe seasonal AR and elevated blood eosinophil counts [15]. In this Special Issue, Sun et al. examined mepolizumab treatment in 60 patients with severe uncontrolled chronic rhinosinusitis with nasal polyps (CRSwNP), a related type 2 inflammatory upper-airway disorder [16]. In this prospective real-world cohort, nasal obstruction and olfactory outcomes improved over six months of add-on treatment with mepolizumab and intranasal corticosteroids, with a favorable safety profile. Evidence across AR and CRSwNP highlights the therapeutic relevance of shared type 2 inflammatory pathways across related upper-airway phenotypes. Comparative studies may clarify how inflammatory phenotype and comorbidity influence biologic response across these disorders.

The contributions to this Special Issue span sensory-neural mechanisms, adaptive immune regulation, molecular heterogeneity, and therapeutic response in AR and related type 2 upper-airway disease. Read alongside the wider literature, they emphasize the value of linking clinical phenotypes with tissue and peripheral immune biology across the course of disease and treatment. Longitudinal studies integrating symptom trajectories, nasal tissue, blood immune phenotypes, and treatment exposure can clarify how these biological states evolve over time, including age-related immune changes during AIT and the reproducibility of molecular stratification across patient populations. Such integrated approaches may also help define patient groups for future evaluation of targeted biologic and experimental cell-based strategies. We thank the authors, reviewers, and the editorial team of *Biomedicines* for their contributions to this Special Issue.
